# Comparison of CIPA Nutritional Screening with GLIM Criteria for Malnutrition, Prognostic Evolution, and Association with Phase Angle in Hospitalized Patients

**DOI:** 10.3390/nu16213652

**Published:** 2024-10-26

**Authors:** Elena Márquez Mesa, Adán Jesús Guerra Cabrera, Ignacio Llorente Gómez de Segura, José Pablo Suárez Llanos

**Affiliations:** 1 Endocrinology and Nutrition Department, Hospital Universitario Nuestra Señora de Candelaria, Ctra. Del Rosario 145, 38010 Santa Cruz de Tenerife, Spainpsuarezllanos@gmail.com (J.P.S.L.); 2 Faculty of Health Sciences, University of La Laguna, Calle Padre Herrera, 38200 La Laguna, Spain

**Keywords:** disease-related malnutrition, CIPA, GLIM, body composition, phase angle, appendicular skeletal muscle index, hospital stay, mortality

## Abstract

Background: Hospital malnutrition has high prevalence and is associated with worse clinical outcomes. The lack of standardized nutritional screening prompted the creation of the CIPA screening tool. Several studies have shown that the phase angle (PA) is associated with increased nutritional risk and worse clinical outcomes. The aim of this study was to establish the concordance between the CIPA and GLIM criteria and to assess their correlation with PA values and clinical outcomes. Methods: A cross-sectional single-center study was carried out, with a prospective six-month follow-up for the prognostic variables. On admission, the CIPA and GLIM criteria and bioimpedanciometry were assessed. Results: A total of 510 inpatients were included; 36.5% had positive CIPA outcomes and 46.1% had positive GLIM outcomes. The correlation between the CIPA and GLIM criteria had a kappa index of 0.26, *p* < 0.01. Those with positive CIPA had a higher mortality risk (OR = 1.81) and longer mean length of stay (MLS) (OR = 1.45). The PA cut-off points were determined by sex and age for CIPA (men > 65 years: 4.75°, men ≤ 65 years: 5.75°, women > 65 years: 4.75°, and women ≤ 65 years: 5.45°) and GLIM (men > 65 years: 4.95°, men ≤ 65 years: 5.85°, women > 65 years: 4.75°, and women ≤ 65 years: 5.55°). These PA cut-off points were associated with worse clinical outcomes with CIPA (mortality OR = 4.2; MLS OR = 1.51; readmissions OR = 2.28) and GLIM (mortality OR = 2.97; MLS OR = 2.61; readmissions OR = 1.79). Conclusions: CIPA screening shows a low correlation with GLIM nutritional assessment. Positive CIPA and GLIM have lower PAs than negative and worse prognostic outcomes. The PA cut-off points associated with worse outcomes have been established.

## 1. Introduction

Malnutrition has a major impact on health outcomes and quality of life for hospitalized patients [1]. Hospitalization carries a high risk for loss of muscle mass and function, which can be accelerated by malnutrition [2]. Hospital malnutrition (HM) is a common problem in hospitalized patients, with prevalence rates ranging from 10 to 50%. In Spain, the multicenter PREDYCES study found that 23.7% of hospitalized patients were undernourished or at risk of undernutrition [3], while the seDREno study, using the GLIM criteria for diagnosis, found a malnutrition prevalence of 29.7% [4]. The early detection of malnutrition and its treatment has been shown to improve the clinical outcomes of these patients [5].

Although malnutrition is well known and widely studied, there is a fundamental lack of consensus regarding the various diagnostic criteria applied in clinical settings, such that no existing approach has ensured wide global acceptance or met the expectations of demographic and etiological differences [6]. For this reason, at the Hospital Universitario Nuestra Señora de Candelaria (HUNSC), a nutritional screening method called CIPA (Control of Intakes, Protein, and Anthropometry) has been developed. It analyzes different items: (a) a decrease in intakes < 50% in the first 72 h of admission; (b) plasma albumin < 3 g/dL; and (c) body mass index (BMI) < 18.5 kg/m^2^ or mid-upper arm circumference (MUAC) ≤ 22.5 cm (in case the BMI cannot be determined). A positive screening is established if at least one of these items is altered, identifying the patient as malnourished or at risk of malnutrition. Since 2015, this screening method has been implemented in the HUNSC, and several validation, optimization, and cost-effectiveness studies have been carried out [7,8].

The development of the GLIM (Global Leadership Initiative on Malnutrition) criteria has led to a common strategy for nutritional assessment, which is performed in two steps: first, a nutritional screening is carried out, and then a nutritional assessment is performed by analyzing phenotypic (nonvoluntary weight loss, low BMI, and reduced muscle mass) and etiological criteria (reduced food intake or absorption and inflammation), thus classifying the severity of malnutrition [9].

On the other hand, the importance of assessing body composition has been increasing in recent years, with bioimpedanciometry (BIA) being the most widely used instrument because it is a non-invasive, low-cost, and accessible method. It is based on the ability of tissues to conduct an electrical current. It measures resistance (R), which corresponds to the opposition of a biological tissue to the flow of an alternating current, and reactance (Xc), which is the tissue’s capacity to store electrical charge like a capacitor. The arc tangent between these variables gives rise to the phase angle (PA). This parameter reflects the integrity of the cell membranes and shows the distribution of water between the intracellular and extracellular space. As opposed to other BIA measurements, PA is obtained without the use of anthropometric parameters. This could be useful in clinical settings, as anthropometry, especially height measurement, is difficult to perform accurately in some patients [10]. Changes in cellularity, cell size, or tissue hydration, lead to variations in the PA. Thus, a low PA suggests cell death and/or decreased cell integrity and may be associated with increased morbidity and mortality in patients [11]. Its role has been investigated as a prognostic marker for mortality in many clinical conditions, such as cancer, kidney and cardiac diseases, people with human immunodeficiency virus, amyotrophic lateral sclerosis, and others, observing that PA has been found to be a predictor of survival [10].

The aim of this study was to establish the concordance between malnutrition or risk of malnutrition as assessed by CIPA and the prevalence of malnutrition as established by the GLIM criteria, and to assess whether malnourished patients or patients at risk of malnutrition have worse PA values and clinical outcomes (mean length of stay, readmission rate, and mortality).

## 2. Materials and Methods

### 2.1. Type of Study and Ethical Aspects

A cross-sectional study was carried out on patients over 18 years of age admitted to the Hospital Universitario Nuestra Señora de Candelaria, assessing the presence of malnutrition or risk of malnutrition using the CIPA nutritional screening tool and GLIM malnutrition criteria, with a prospective follow-up after 6 months to evaluate the prognostic variables. The HUNSC ethics committee approved this study on 17 December 2020 (project code CHUNSC-2020-105). This study was conducted in accordance with the requirements expressed in the Declaration of Helsinki (Fortaleza (Brazil) review, October 2013) and the laws in force in Europe and Spain. An information sheet was given to the participants. The investigator explained to each patient the objectives and procedures of the study and requested the informed consent document to be signed. Once the consent form was signed, the researcher began the explorations and data collection necessary for this study. The investigator did not conduct any research for this study until consent was obtained from the patient. A total of 510 patients (270 men/240 women) were included. The most frequent admission services were internal medicine (12.7%), traumatology (12.7%), digestive (12.4%), nephrology (10.6%), and neurology (10%).

### 2.2. Inclusion and Exclusion Criteria

Patients over 18 years of age of both sexes and with an average hospital stay of more than 3 days were included. Patients admitted to departments with a low prevalence of malnutrition (ophthalmology, dermatology, obstetrics, etc.); pediatric, intensive care, and palliative care patients; or patients receiving artificial nutrition (oral nutritional supplements, enteral nutrition, or parenteral nutrition) were excluded. Likewise, patients with fluid overload were evaluated by their attending physician, and those in whom it was individually considered that this degree of edema could affect the results of the BIA measurement were excluded.

Written informed consent was obtained from the patients who met all the inclusion criteria and none of the exclusion criteria. In the case of disabled patients, consent was obtained from their legal guardians.

### 2.3. Data Collection

The CIPA nutritional screening tool was performed as usual in the HUNSC, and the GLIM criteria were subsequently applied. The nutritional assessment was carried out after the third day of admission until the fifth day of admission (with no more than 2 days between the nutritional screening and the nutritional assessment). The CIPA and GLIM results were obtained from the clinical history and interviews, conducted by a physician specializing in endocrinology and nutrition and assisted by a medical student. For CIPA, the BMI, plasma albumin levels, and percentage reduction in oral intake of the diet prescribed by their attending physician were determined. The plasma albumin was determined by the bromocresol green method, and the health workers who collected each meal marked on a document whether they ate more or less than 50%. Positivity for at least one of these items resulted in a positive screening: (a) decreased intake of <50% in the first 72 h of admission; (b) plasma albumin of < 3 g/dL; and (c) body mass index (BMI) of <18.5 kg/m^2^ or mid-upper arm circumference (MUAC) of ≤ 22.5 cm (in case the BMI could not be determined).

For a diagnosis of malnutrition using the GLIM criteria, at least one phenotypic and one etiological criterion was required to be altered. For the phenotypic criteria, we determined the following: unintentional weight loss of ≥5–10% in the last 6 months; BMI of <20 kg/m^2^ in <70 years and <22 kg/m^2^ in >70 years. The appendicular skeletal muscle mass index (ASMI = ASM/height^2^) was defined as altered if below 7 kg/m^2^ in men and 5.5 kg/m^2^ in women. For the etiological criteria, the presence of decreased intake was established if these were <50% for 1 week or if there was any decrease in intake for 2 weeks, as well as the presence of gastrointestinal disturbances justifying a decrease in nutrient absorption. The presence of inflammation was assessed by the presence of acute/chronic disease, as well as C-reactive protein determination by immuno-turbidimetry.

The PA and ASMI were assessed by electrical bioimpedance (BIA 101^®^ Akern Anniversary, Akern SRL, Pontassieve, Florence, Italy).

In addition, variables such as age, sex, cause of admission, and comorbidities (Charlson comorbidity index (CCI)) were collected. Subsequently, the patients with positive CIPA received the nutritional intervention established in the HUNSC protocol (two specific oral nutrition supplements per day, depending on their comorbidities, and dietary adjustment managed by a dietitian).

The patients were followed up for 6 months to evaluate the prognostic variables (mean length of stay, readmission rate, and mortality). Patients were included in the period between February 2021 and September 2023.

### 2.4. Statistical Analysis

The qualitative variables were summarized as frequency distributions, and the normally distributed quantitative variables as the means ± standard deviations (SDs). The continuous, non-normally distributed variables were summarized as the medians and interquartile ranges (IQRs).

The associations among the qualitative variables were determined using the chi-square test. The means were compared between two independent groups using the parametric Student’s *t*-test or the nonparametric Mann–Whitney U test for non-normally distributed variables.

To evaluate the diagnostic capacity of CIPA for the diagnosis of malnutrition (using the GLIM criteria as the gold standard), the sensitivity, specificity, and positive and negative predictive values were calculated. The concordance between the GLIM and CIPA was studied using Cohen’s kappa index.

The relationships between the outcome variables of 6-month mortality, readmission for 30 days, and hospital length of stay (≥15 days) and the diagnostic criteria of CIPA and GLIM were assessed using binary logistic regression adjusted by age, sex, CCI, and department of admission.

The receiver operating characteristic (ROC) curve and the area under the ROC curve (AUC) were used to determine the cut-off values of the phase angle, according to age and gender, that indicated the presence of malnutrition for the CIPA and GLIM criteria. The cut-off values were determined using the index of union (IU). Once the cut-off points were established, they were related to the outcome variables by means of adjusted logistic regression models, as explained above. Statistical significance was assumed as *p* < 0.05. All analyses were performed using SPSS 26.0 (IBM Corp., Armonk, NY, USA).

## 3. Results

### 3.1. Baseline Characteristics of the Sample

A total of 510 patients who met the inclusion criteria during the data collection period agreed to participate in the study. Of the admissions, 72.4% were in medical services, and 27.6% were in surgical services. In total, 52.9% were male, the mean age was 65.3 ± 14.86 years, 83.7% had CCI > 3, and the mean PA was 5.44 ± 1.68°. Table 1 shows the baseline characteristics.

### 3.2. Screening and Diagnosis of Malnutrition

Positive CIPA outcomes were determined in 36.5% of the sample. Table 2 shows the results of the variables assessed. The CIPA outcomes were positive for one altered item in 29% of the patients, two items in 6.5%, and three items in 1%.

Malnutrition was found in 46.1% of the sample by application of the GLIM criteria. A total of 61.6% were positive for the phenotypic criteria and 70.2% for the etiological criteria. Table 2 shows the results of the different criteria evaluated.

Assuming the GLIM criteria as the reference method for nutritional assessment, the study of the diagnostic ability of CIPA showed a sensitivity of 50.2%, specificity of 75.3%, PPV of 63.4%, NPV of 63.9%, AUC of 0.63 (CI 0.59–0.67), and kappa index of 0.26 (*p* < 0.01). Table 3 shows the ratios of positive and negative CIPA and GLIM outcomes and Table 4 shows the baseline characteristics of patients according to the presence of malnutrition or risk of malnutrition as determined by CIPA screening and GLIM criteria

### 3.3. Association of CIPA and Positive GLIM Criteria with Clinical Course

The positive CIPA patients had higher mortality than the negative patients (26.9% vs. 14.5%, *p* = 0.001) and a mean length of stay of > 15 days (53.2% vs. 44.1%, *p* = 0.048). There was a trend toward a higher rate of early readmission in the positive CIPA patients (16.7% vs. 12%, *p* = 0.144), although this was not significant.

**Figure 1:**
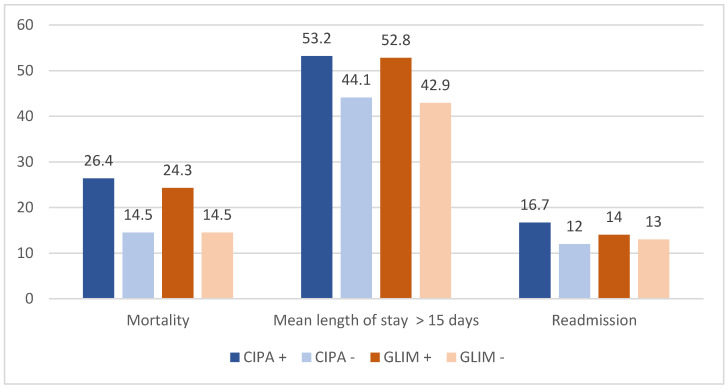
Percentages of mortality, mean length of stay, and readmissions by group.

With respect to the positive GLIM patients, higher mortality and a mean length of stay of > 15 days were also observed (24.3% vs. 14.5%, *p* = 0.005 and 52.8% vs. 42.9%, *p* = 0.026, respectively), but no higher risk of early readmission was determined (14% vs. 13.5%, *p* = 0.847). See Figure 1.

Table 5 shows the results of the multivariate analysis of the relationship between the CIPA and GLIM criteria, with the prognostic variables adjusted for age, sex, CCI, and admission service (medical or surgical) showing higher mortality and a longer mean length of stay for CIPA (OR = 1.81, *p* 0.019 and OR = 1.45, *p* 0.049, respectively) and a trend of higher mortality for GLIM (OR = 1.51, *p* 0.075).

Regarding the ability of CIPA and GLIM to predict 6-month mortality, AUCs of 0.59 and 0.58 were determined, respectively (*p* = 0.668). For a mean length of stay of > 15 days, AUCS of 0.54 and 0.55 (*p* = 0.795), and for early readmission, AUCs of 0.54 and 0.51 (*p* = 0.349) were determined, respectively.

### 3.4. Association of CIPA and GLIM with PA

The mean PA in positive CIPA patients was 5 ± 1.51° vs. 5.7 ± 1.72° in the negative CIPA patients (*p* < 0.01), while PAs of 5.15 ± 1.51° vs. 5.7 ± 1.78° for the positive GLIM vs. the negative GLIM patients (*p* < 0.01) were determined. Regarding gender distribution, the mean PA for men was 5.57 ± 1.59° vs. 5.29 ± 1.78° for women (*p* = 0.059). Patients >65 years had a mean PA of 5.07 ± 1.5° vs. 5.86 ± 1.77° in those <65 years (*p* < 0.01). Table 6 shows the mean PAs by sex and age group according to the results obtained from CIPA and GLIM.

### 3.5. CIPA and GLIM Association with PA and Prognostic Outcomes

Table 6 shows the results of the ROC curve analysis. A moderate predictive ability was found for the PA and nutritional risk determined with CIPA in men of all ages (>65 years, AUC of 0.644, 95% CI: 0.55–0.74; <65, AUC of 0.743, 95% CI: 0.65–0.83) and women >65 years (AUC of 0.689, 95% CI: 0.59–0.79). However, the predictive ability for malnutrition by PA decreased for the GLIM criteria. The PA cut-off points for discriminating nutritional risk and undernutrition by CIPA and GLIM can be seen in Table 7.

Table 8 shows the results of the multivariate analysis of the association of these PA cut-off points for CIPA and GLIM with the prognostic variables (adjusted for age, sex, CHF, and admission service (medical or surgical)), with significantly higher mortality, mean lengths of stay, and readmission rates for both.

## 4. Discussion

This study evaluated the clinical prognosis of patients with malnutrition and risk of malnutrition as determined by CIPA nutritional screening and the GLIM criteria. In addition, we studied whether malnourished patients had worse PAs, establishing cut-off points for malnutrition detection and analyzing their prognostic value.

A 36.5% prevalence of malnutrition or risk of malnutrition was determined by CIPA, similar to the rates described in previous studies in which this screening tool has been applied, with 35.8% [12] and 35.4% [7]. However, this prevalence was higher than that described in the PREDYCES study (23.7%) [3], using the nutritional risk screening tool (NRS 2002), although it was 37% in those over 70 years of age, which is closer to the patients included in our study. On the other hand, using the GLIM criteria, a prevalence of malnutrition of 46.1% was detected, which is higher than that found in the seDREno study (29%) [4] with the application of the same criteria, although it also increased in the group over 70 years of age to 34.8%, similar to the rates described by Allard et al. (33.3%) [13] and Dronkelaar et al. (42%) [14]. It should be taken into consideration that the patients included in our study had a high rate of comorbidities, which could justify the higher prevalence compared to the other studies in which GLIM criteria have been used. However, previous studies have reported a higher prevalence of malnutrition with the application of the GLIM criteria compared to other screening or nutritional assessment tools [15,16,17,18].

Regarding the diagnostic ability of CIPA for malnutrition using the GLIM criteria as a reference, a PPV of 63.4%, an AUC of 0.63 (CI: 0.59–0.67), and a kappa index of 0.26 (*p* < 0.01) indicate a low diagnostic ability and correlation between the tools. Similar correlations between GLIM and other nutritional screening tests have also been described in previous studies: Boulhosa et al. compared it to the NRS-2002 (kappa index of 0.43 and AUC of 0.731) [19]; Dronkelaar et al. [14] compared it to the Short Nutritional Assessment Questionnaire (SNAQ) (kappa index of 0.57), the Malnutrition Universal Screening Tool (MUST) (kappa index of 0.39), the Malnutrition Screening Tool (MST) (kappa index of 0.59), the Mini Nutritional Assessment-Short Form (MNA-SF) (kappa index of 0.21), and the Patient-Generated Subjective Global Assessment-Short Form (PG-SGA-SF) (kappa index of 0.29); and Clark et al. compared it to the MST (AUC 0.63, kappa index of 0.26) [16]. When analyzing these data, it is necessary to highlight the importance of taking into account that we did not employ two equal tools, but rather we compared a nutritional screening and a diagnostic nutritional assessment, with the comparison of nutritional risk not equivalent to the diagnosis of malnutrition [6].

In terms of prognostic outcomes, higher mortality and a longer mean length of stay were found for the positive CIPA patients, and a trend toward higher mortality and a longer mean length of stay were found for the positive GLIM patients. These results are similar to those obtained in previous studies, in which the presence of malnutrition has been associated with worse clinical outcomes [4,7,20,21].

PA is an indicator of health and nutritional risk in hospitalized patients, reflecting both cell membrane integrity and intracellular water distribution [22]. Although PA has been used as a clinical prognostic tool, it is important to establish cut-off points that allow for the identification of patients admitted at increased nutritional risk to optimize their clinical management. Cut-off points for detecting malnutrition vary from 4.73–6° in different studies [23,24,25,26]. In our study, cut-off points that varied between 4.75 and 5.85° were established by age group and sex for malnutrition or nutritional risk as determined by CIPA and GLIM, with slightly lower cut-off points for CIPA, which could suggest that this tool detects patients with greater impairment of cellular integrity compared to GLIM.

Regarding the prognostic outcomes, it has been shown that the established PA cut-off points were associated with higher mortality in both CIPA and GLIM evaluation. These results are consistent with those obtained in previous studies that have reported that a lower PA is associated with higher mortality in different pathologies [10]. For example, a study in patients with COVID-19 found that a PA cut-off point of 3.95° was predictive of higher 90-day mortality [27], and Saueressig et al. [28] described in patients with decompensated cirrhosis that each 1° increase in the PA was associated with a 53% decrease in 6-month mortality. Likewise, the cut-off points obtained for both tests were associated with a longer average length of stay and higher readmission rates.

As the limitations of the study, it should be noted that this was a single-center study, and the data should be extrapolated with caution to the general population. In our study, the body composition assessment was performed with the BIA 101^®^ Akern Anniversary bioimpedancemeter, and the results may differ from those performed with other devices. It was not recorded which patients received nutritional or rehabilitation therapy, so it was not possible to evaluate whether those who were treated had a better prognostic outcome.

## 5. Conclusions

In summary, our study shows a low correlation between CIPA screening and GLIM nutritional assessment. The positive CIPA patients showed worse prognostic outcomes, with higher mortality and a longer mean length of stay, while the positive GLIM patients showed a trend toward worse prognostic outcomes but without reaching statistical significance. The positive CIPA and GLIM patients had lower PAs than the negative patients, and the PA cut-off points established for both tests were associated with worse clinical outcomes (mortality, mean length of stay, and readmission rate). In view of these results, it is of interest to assess the PA in patients at nutritional risk for the early and easy detection of those who may have worse clinical outcomes.

## Figures and Tables

**Table 1:** Baseline characteristics of the sample.

	*n* = 510 Mean (SD)
Age (years)	65.3 (14.86)
Sex (% men)	52.9
CCI	7.64 (5.27)
BMI (kg/m^2^)	26.93 (6.61)
ASMI (kg/m^2^)	7.25 (1.71)
Albumin (g/dL)	3.52 (0.63)
PA (°)	5.44 (1.68)

SD = standard deviation. CCI = Charlson comorbidity index. BMI = body mass index. ASMI = appendicular skeletal muscle mass index. PA = phase angle.

**Table 2:** Results obtained from the evaluation of the CIPA nutritional screening and the GLIM criteria.

CIPA Screening	*n* = 510 (%)	GLIM Criteria		*n* = 510 (%)
Plasma albumin	113 (22.2)	**Phenotypic criteria** 314 (61.6)	Unintentional weight loss	245 (48)
Decreased intakes	85 (16.7)		BMI	82 (16.1)
BMI	31 (6.1)		Decrease in ASMI	147 (28.8)
		**Etiological criteria** 358 (70.2)	Decreased intake	85 (16.7)
			Inflammation	336 (65.9)
CIPA positive 186 (36.5)		GLIM positive 235 (46.1)		

BMI = body mass index. ASMI = appendicular skeletal muscle mass index.

**Table 3:** Ratios of positive and negative CIPA and GLIM outcomes.

	CIPA +	CIPA –	Total
**GLIM +**	118	117	235
**GLIM –**	68	207	275
**Total**	186	324	510

**Table 4:** The baseline characteristics of patients according to the presence of malnutrition or risk of malnutrition as determined by CIPA screening and GLIM criteria.

		CIPA +	CIPA −	*p*	GLIM +	GLIM −	*p*
Sex	Men, *n* (%)	100 (53.8)	170 (52.5)	0.778	139 (59.2)	131 (47.6)	<0.01
	Women, *n* (%)	86 (46.2)	154 (47.5)		96 (40.9)	144 (52.4)	
Age	>65 years, *n* (%)	104 (55.9)	165 (50.9)	0.277	144 (61.3)	125 (45.5)	<0.01
	<65 years, *n* (%)	82 (44.1)	159 (49.1)		91 (38.7)	150 (54.5)	
Admission service	Medical, *n* (%)	141 (75.8)	228 (70.4)	0.186	155 (66)	214 (77.8)	<0.01
	Surgical, *n* (%)	45 (24.2)	96 (29.6)		80 (34)	61(22.2)	
Type of admission	Urgent, *n* (%)	170 (91.4)	228 (88.9)	0.367	209 (88.9)	249 (90.5)	0.549
	Programmed, *n* (%)	16 (8.6)	36 (11.1)		26 (11.1)	26 (9.5)	
CCI	0–1, *n* (%)	9 (4.8)	29 (9)	0.067	12 (5.1)	26 (9.5)	0.025
	2, *n* (%)	12 (6.5)	33 (10.2)		15 (6.4)	30 (10.9)	
	≥3 (%)	165 (88.7)	262 (80.9)		208 (88.5)	219 (79.6)	
BMI (kg/m^2^), mean (SD)		25 (6.76)	28.03 (6.27)	<0.01	25.1 (6.01)	28.49 (6.71)	<0.01
Albumin (g/dL), mean (SD)		3.07 (0.64)	3.79 (0.45)	<0.01	3.38 (0.63)	3.64 (0.61)	<0.01
ASMI (kg/m^2^), mean (SD)		7.04 (1.81)	7.37 (1.64)	0.043	6.82 (1.72)	7.62 (1.63)	<0.01

CCI = Charlson comorbidity index. BMI = body mass index. ASMI = appendicular skeletal muscle mass index.

**Table 5:** Association of CIPA and GLIM criteria with worse prognostic outcomes (mortality, mean length of stay, and readmissions).

	Mortality (<6 Months)		Mean Length of Stay (>15 Days)		Readmissions (<30 Days)	
	OR_a_ (IC 95%)	*p*	OR_a_ (IC 95%)	*p*	OR_a_ (IC 95%)	*p*
**CIPA +**	1.81 (1.15–2.98)	0.019	1.45 (1.00–2.09)	0.049	1.23 (0.72–2.10)	0.440
**GLIM +**	1.51 (0.95–2.62)	0.075	1.31 (0.91–1.89)	0.142	0.91 (0.53–1.56)	0.730

OR_a_ (IC) = adjusted odds ratio (confidence interval). Multivariate analysis adjusted for age, sex, CCI, and admission service (medical/surgical).

**Table 6:** PAs by sex and age according to CIPA and GLIM results.

Sex	Age	CIPA +	CIPA −	*p*	GLIM +	GLIM −	*p*
Men	>65 years	4.97 (1.95)	5.41 (1.49)	0.123	5.09 (1.72)	5.45 (1.62)	0.206
	≤65 years	5.24 (1.4)	6.38 (1.18)	<0.01	5.68 (1.45)	6.2 (1.28)	0.038
Women	>65 years	4.79 (1.3)	4.92 (1.22)	0.580	4.68 (1.2)	5.05 (1.25)	0.092
	≤65 years	5.04 (1.19)	6.08 (2.36)	0.015	5.27 (1.29)	5.96 (2.36)	0.114

**Table 7:** Prognostic ability of PA cut-off points for undernutrition determined by CIPA and GLIM.

CIPA	Age	AUC	95% CI	*p*	Cut-Off Point (°)	Sensibility (%)	Specificity (%)
Men	>65 years	0.644	0.55–0.74	<0.01	≤4.75	57.4	59.3
	≤65 years	0.743	0.65–0.83	<0.01	≤5.75	65.2	70.9
Women	>65 years	0.536	0.43–0.64	0.492	≤4.75	58	52.7
	≤65 years	0.689	0.59–0.79	<0.01	≤5.45	63.9	65
**GLIM**	**Age**	**AUC**	**95%** **CI**	***p***	**Cut-Off Point (°)**	**Sensibility (%)**	**Specificity (%)**
Men	>65 years	0.582	0.49–0.67	0.091	≤4.95	56.1	57.1
	≤65 years	0.610	0.51–0.71	0.035	≤5.85	56.1	55.9
Women	>65 years	0.595	0.49–0.7	0.067	≤4.75	58.1	54.8
	≤65 years	0.602	0.49–0.72	0.084	≤5.55	58.8	53.7

AUC = area under receiver operating curve; CI = confidence interval.

**Table 8:** Association of PA cut-off points for CIPA and GLIM criteria with worse prognostic evolution (mortality, mean length of stay, and readmissions).

	Mortality (<6 Months)		Mean Length of Stay (>15 Days)		Readmissions (<30 Days)	
	OR_a_ (CI 95%)	*p*	OR_a_ (CI 95%)	*p*	OR_a_ (CI 95%)	*p*
Cut-off point, CIPA	4.14 (2.4–7.15)	<0.01	1.95 (1.3 7–2.83)	<0.01	1.84 (1.07–3.15)	0.027
Cut-off point, GLIM	4.28 (2.43–7.55)	<0.01	2.11 (1.47–3.03)	<0.01	1.79 (1.04–3.11)	0.036

OR_a_ (CI) = adjusted odds ratio (confidence interval). Multivariate analysis adjusted for age, sex, CCI, and admission service (medical/surgical).

## Data Availability

The original contributions presented in the study are included in the article, further inquiries can be directed to the corresponding author.
